# The Physician’s Visiting List, Diary, and Book of Engagements, for 1853

**Published:** 1852-10

**Authors:** 


*The Physician's Visiting List, Diary, and Book of Engagements, for* 1853. Philadelphia, Lindsay & Blakiston, 25 South Sixth Street.

Our prognostications in relation to this most acceptable offering to the profession, were speedily realized in the sale of the edition of 1852, and it has proved itself to be one of those conveniences which no man will ever be without, when once discovered.

We have received from the publishers a copy of the Visiting

List for 1853. Besides space for recording twenty-five visits per diem, it contains blanks for memoranda, obstetric engagements, residence of nurses, vaccination engagements, list of things lent to patients and others, accounts asked for, memoranda, a table of poisons and their antidotes, a table of doses, proceedings of the American Medical Association, and an excellently arranged almanac, and the whole is conveniently arranged so as to be readily carried in the pocket. From abundant experience we warmly commend it to our readers both in the town and in the country.

